# Drug-resistance in doxorubicin-resistant FL5.12 hematopoietic cells: elevated MDR1, drug efflux and side-population positive and decreased BCL2-family member expression

**DOI:** 10.18632/oncotarget.22956

**Published:** 2017-12-06

**Authors:** Linda S. Steelman, Steve L. Abrams, Peter Ruvolo, Vivian Ruvolo, Lucio Cocco, Stefano Ratti, Alberto M. Martelli, Luca M. Neri, Saverio Candido, Massimo Libra, James A. McCubrey

**Affiliations:** ^1^ Department of Microbiology and Immunology, Brody School of Medicine at East Carolina University, Greenville, SC, USA; ^2^ Section of Signal Transduction and Apoptosis, Hormel Institute, University of Minnesota, Austin, TX, USA; ^3^ Current/Present address: Department of Leukemia, University of Texas MD Anderson Cancer Center, Houston, TX, USA; ^4^ Dipartimento di Scienze Biomediche e Neuromotorie, Università di Bologna, Bologna, Italy; ^5^ Department of Morphology, Surgery and Experimental Medicine, University of Ferrara, Ferrara, Italy; ^6^ Department of Biomedical and Biotechnological Sciences – Oncological, Clinical and General Pathology Section, University of Catania, Catania, Italy

**Keywords:** drug transporters, bcl-2, MEK1, p53, cancer stem cells

## Abstract

Chemotherapeutic drug treatment can result in the emergence of drug-resistant cells. By culturing an interleukin-3 (IL-3)-dependent cell line, FL5.12 cells in the presence of the chemotherapeutic drug doxorubicin, we isolated FL/Doxo cells which are multi-drug resistant. Increased levels of drug efflux were detected in FL/Doxo cells which could be inhibited by the MDR1 inhibitor verapamil but not by the MRP1 inhibitor MK571. The effects of TP53 and MEK1 were examined by infection of FL/Doxo cells with retroviruses encoding either a dominant negative TP-53 gene (FL/Doxo+ TP53 (DN) or a constitutively-activated MEK-1 gene (FL/Doxo + MEK1 (CA). Elevated MDR1 but not MRP1 mRNA transcripts were detected by quantitative RT-PCR in the drug-resistant cells while transcripts encoding anti-apoptotic genes such as: BCL2, BCLXL and MCL1 were observed at higher levels in the drug-sensitive FL5.12 cells. The percentage of cells that were side-population positive was increased in the drug-resistant cells compared to the parental line. Drug-resistance and side-positive population cells have been associated with cancer stem cells (CSC). Our studies suggest mechanisms which could allow the targeting of these molecules to prevent drug-resistance.

## INTRODUCTION

The PI3K/PTEN/AKT/mTORC1/GSK-3, RAS/RAF/MEK1/ERK, and TP53 pathways are critical in many biological processes including cancer progression and drug resistance. These pathways also exert pivotal roles in cell growth, death and senescence. The biological and biochemical effects of the Raf/MEK1/ERK, PI3K/PTEN/AKT/mTORC1/GSK-3 and TP53 pathways have been recently reviewed [1–7]. These signaling cascades are also important in cardiovascular diseases, diabetes, metabolism, neurological diseases, obesity and other maladies [8–10]. A key factor controlled by both pathways positively and negatively is the mTORC complex which is critical in regulating protein translation and is often dysregulated in various diseases [2, 4, 5, 7].

Dysregulation of the PI3K/PTEN/Akt/mTORC1/GSK-3 pathway often occurs in human cancer. Certain members are thought of as oncogenes *e.g*., PI3K, AKT and GSK-3, while other members are considered to be tumor-suppressor genes *e.g*., PTEN and GSK-3 [11–30]. It is noted that GSK-3 can function as a tumor suppressor or an oncogene which might be due to the cell type or biological situation [1–6].

Inhibition of the PI3K/PTEN/AKT/mTORC/70S6K pathway by protein interacting with carboxyl terminus 1 (PICT-1, a.k.a NOP53 ribosome biogenesis factor) can result in autophagy which results in cell death [31]. Serum-and glucocorticoid-induced protein kinase (SGK1) is related to AKT. Inhibition of SGK1 in glioblastoma cells can lead to cytotoxic autophagy [32]. Autophagy is a key process controlling cell death, cell fate and sensitivity to chemotherapeutic drugs, as well as, cancer [33–40].

The RAS/RAF/MEK/ERK pathway also consists of proteins which are considered as oncogenes, *e.g*., RAS, RAF and other proteins which may have tumor suppressor activities *e.g*., protein phosphatase 2A (PP2A), dual specificity protein phosphatase 1 (DUSP1) and others. The altered expression of components of this pathway can result in resistance to targeted therapeutics [33].

The TP53 pathway is critical in controlling many biological processes such as division, senescence, quiescence, geroconversion, autophagy, drug resistance and cancer induction [34–44]. Activation of autophagy can contribute to chemotherapeutic drug resistance [45]. The TP53 protein regulates many events and is involved in the regulation of microRNAs (miRs). The TP53 gene is frequently mutated in human cancer. In some cases, the mutations result in novel TP53 molecules which have gain of function activity [46].

Various classes of proteins are frequently implicated in resistance to chemotherapeutic drugs as well as targeted therapeutics. Prominent classes include proteins involved in signal transduction such as PI3K/PTEN/AKT/mTORC/GSK-3, RAF/MEK/ERK, TP53 and BCL2 pathways [47–51]. The BCL2 family and other molecules which regulate apoptosis are also frequently deregulated in cancer and drug resistance [52–54]. The activity and levels of apoptotic regulatory proteins are often themselves regulated by the PI3K/PTEN/Akt/mTORC/GSK-3, Raf/MEK/ERK, TP53 and other pathways by phosphorylation and transcriptional mechanisms [55–61].

Proteins that are frequently referred to as “drug-transporters” are often upregulated in drug-resistant cells [62–64]. The “drug-transporting” functions of these proteins are often considered a “moon-lighting” activity of these proteins as they often have physiological roles in various organs (*e.g*., gut, colon) to transport xenobiotics [65–67].

Increased expression of MDR1 and/or MRP1 has been observed in certain drug-resistant hematopoietic cells such as HL-60 [68]. The MDR1 (a.k.a., Pgp, ABCB1) has been shown to efflux various chemotherapeutic drugs such as doxorubicin, daunorubicin, paclitaxel and others [69]. MRP1 (a.k.a. ABCC1) will transport various molecules including: daunorubicin, doxorubicin, edatrexate, etoposide, epirubicin, idarubicin, irinotecan, methotrexate, paclitaxel, vinblastine, vincristine and others [70]. Drug transporters may in some cases be regulated by miRs which in turn will change the sensitivity of certain tumors to chemotherapeutic drugs [71].

FL5.12 is an interleukin-3 (IL-3)-dependent hematopoietic cell line derived from the fetal liver of inbred mice [72]. They absolutely require IL-3 for their survival and after 18-24 hours of IL3-deprivation they commence to undergo apoptosis [73]. The IL3-dependency of these cells can be relieved after introduction of various activated oncogenes either by themselves (v-ABL, BCR-ABL) [74] or in combination (AKT and RAF) [75]. In addition, activated cytokine genes such as IL-3 genes with mutations in the 3'untranslated region in the sequence motifs associated with mRNA (in)stability will relieve the dependency of these cells on the addition of exogenous IL-3 [76, 77]. FL5.12 cells do not normally cause tumors upon injection into nude immunocompromised mice, however, once their dependency on addition of exogenous IL-3 is abrogated, they usually will form tumors in immunocompromised mice [77, 78].

We derived a panel of doxorubicin-resistant FL5.12 cells (FL/Doxo) by culturing them in the presence of doxorubicin for prolonged time periods [79]. These cells have been determined to have altered TP53 and ERK signaling. Furthermore, these cells show an altered response to certain small molecule inhibitors which target various molecules such as the mTORC1 complex [80]. We have previously shown that the doxorubicin-resistant FL/Doxo cells are cross resistant to daunorubicin, paclitaxel but not 5-fluorouracil (5FU) [79]. We demonstrated recently that increased levels of mRNA transcripts encoding MDR1 but not MRP1 were detected by q-RT-PCR in FL/Doxo in comparison to FL5.12 cells [80]. The drug-resistance of the FL/Doxo cells could be increased further by infection of the cells with retroviruses which encode either dominant negative (DN) TP53 (FL5.12 cells have wild-type TP53) or constitutively-active (CA) MEK1 [79].

In the following studies, the drug efflux abilities of doxorubicin-resistant FL/Doxo and doxorubicin-sensitive FL5.12 cells were determined, as well as, the presence of side-population positive cells in both types of cells. We document the effects of TP53(DN) and MEK1(CA) oncoproteins on drug-resistance and side-population positive cells. These studies provide insights into mechanisms responsible for drug-resistance and side-positive population cells and may indicate approaches to target these cells which are often responsible for cancer development and progression.

## RESULTS

### IL-3 suppresses effects of doxorubicin in parental FL5.12 cells

**Figure 1:**
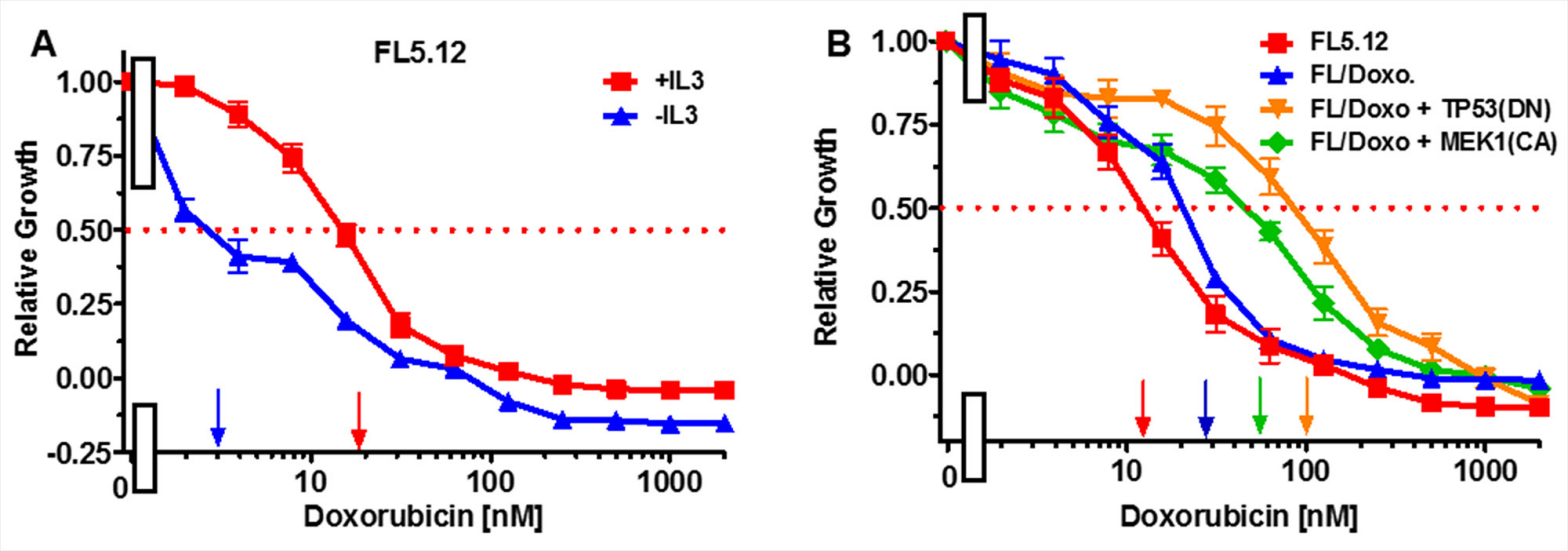
Effects of IL-3 on Sensitivity of FL5.12 cells to doxorubicin and sensitivies of FL5.12 and FL/Doxo cells to doxorubicin **(Panel A)** The effects of IL-3 (solid red squares) and the absence of IL-3 (solid blue triangles) on the sensitivities of FL5.12 cells to different concentrations doxorubicin and then examined by the 2-yl)-2,5-diphenyl tetrazolium bromide (MTT) assay as described [79, 80]. The MTT assay is a colorimetric assay that measures the reduction of MTT by mitochondrial succinate dehydrogenase as described [80]. Arrows pointing to the X-axis indicate where the IC_50_ can be estimated. Statistical analysis (unpaired *t* test results) indicated that the two-tailed *P* value: for (Panel A) FL5.12 in the presence of IL-3 and FL5.12 in the absence of IL-3 is less than 0.0001 which is considered to be extremely statistically significant. **(Panel B)** The effects of doxorubicin on: FL5.12 (solid red squares), FL/Doxo (solid blue upward triangles), FL/Doxo + TP53 (DN) (solid orange downward diamonds) and FL/Doxo + MEK1 (CA) (solid green diamonds) cells were determined by titrating all the cells on the same day with in the presence of IL-3 and then examined four days by MTT analysis. The *P* values for comparisons of FL5.12, FL/Doxo, FL/Doxo + TP53(DN) and FL/Doxo + MEK1(CA) are less than 0.0001 which are considered to be extremely statistical significant. These experiments were performed three times with similar results.

The effects of doxorubicin were examined on the cytokine-dependent FL5.12 cells. When these cells were cultured in the presence of IL-3, the IC_50_ for doxorubicin was approximately 20 nM (Figure 1, Panel A). In contrast, when the cells were cultured in the absence of IL-3, the level of growth decreased and the IC_50_ for doxorubicin was approximately 3 nM (Figure 1, Panel A) which was 6.7-fold lower than when the cells were cultured in IL-3.

### Doxorubicin-resistance of FL/Doxo and derivative cells

The doxorubicin-sensitivities of the FL5.12, FL/Doxo, FL/Doxo + TP53(DN) and FL/Doxo + MEK1(CA) cells are presented in Figure 1, Panel B. In this experiment the doxorubicin IC_50_s for: FL5.12, FL/Doxo, FL/Doxo + TP53(DN) and FL/Doxo + MEK1(CA) cells were approximately 12, 30, 100 and 55 nM respectively. These IC_50_s were all measured on the same day. There is some variation in IC_50_s measured on different days that is why it is better to make comparisons on results obtained on the same day.

### Drug-resistant FL/Doxo cells are frequently larger cells than drug-sensitive FL5.12 cells

**Figure 2:**
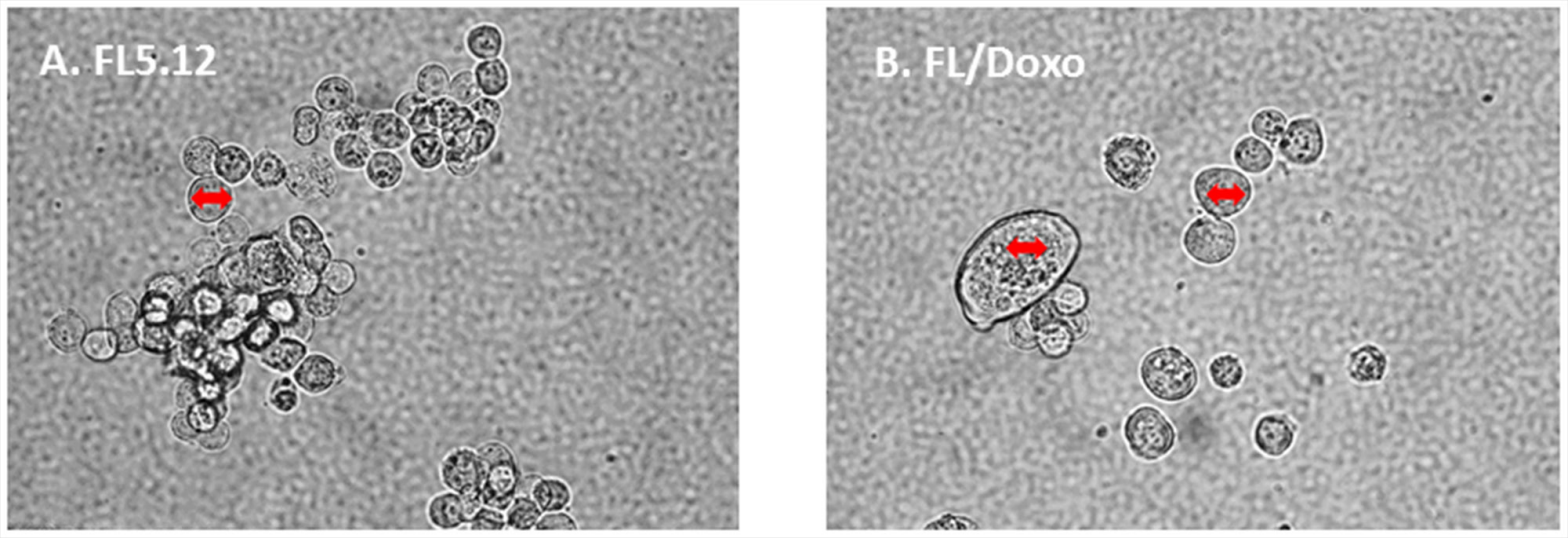
Morphology of FL5.12 and FL/Doxo cells The morphologies of **(Panel A)** FL5.12 and **(Panel B)** FL/Doxo cells were examined by phase microscopsy. The double-sided arrow in the cells is the same size in the different cells and serves to visualize how much larger some of the FL/Doxo cells are in comparison to the FL5.12 cells.

The morphologies of the FL5.12 and FL/Doxo cells were examined by phase contrast microscopsy (Figure 2). FL/Doxo cells (Panel B) were larger than parental FL5.12 cells (Panel A). Often there were “giant” cells present in the FL/Doxo cultures [81]. Large multi-nucleate cells have been observed with drug-resistant prostate cancer cells [82].

### Increasing concentrations of doxorubicin result in more apoptotic cells in drug-sensitive FL5.12 than other drug-resistant FL5.12 cells

**Figure 3:**
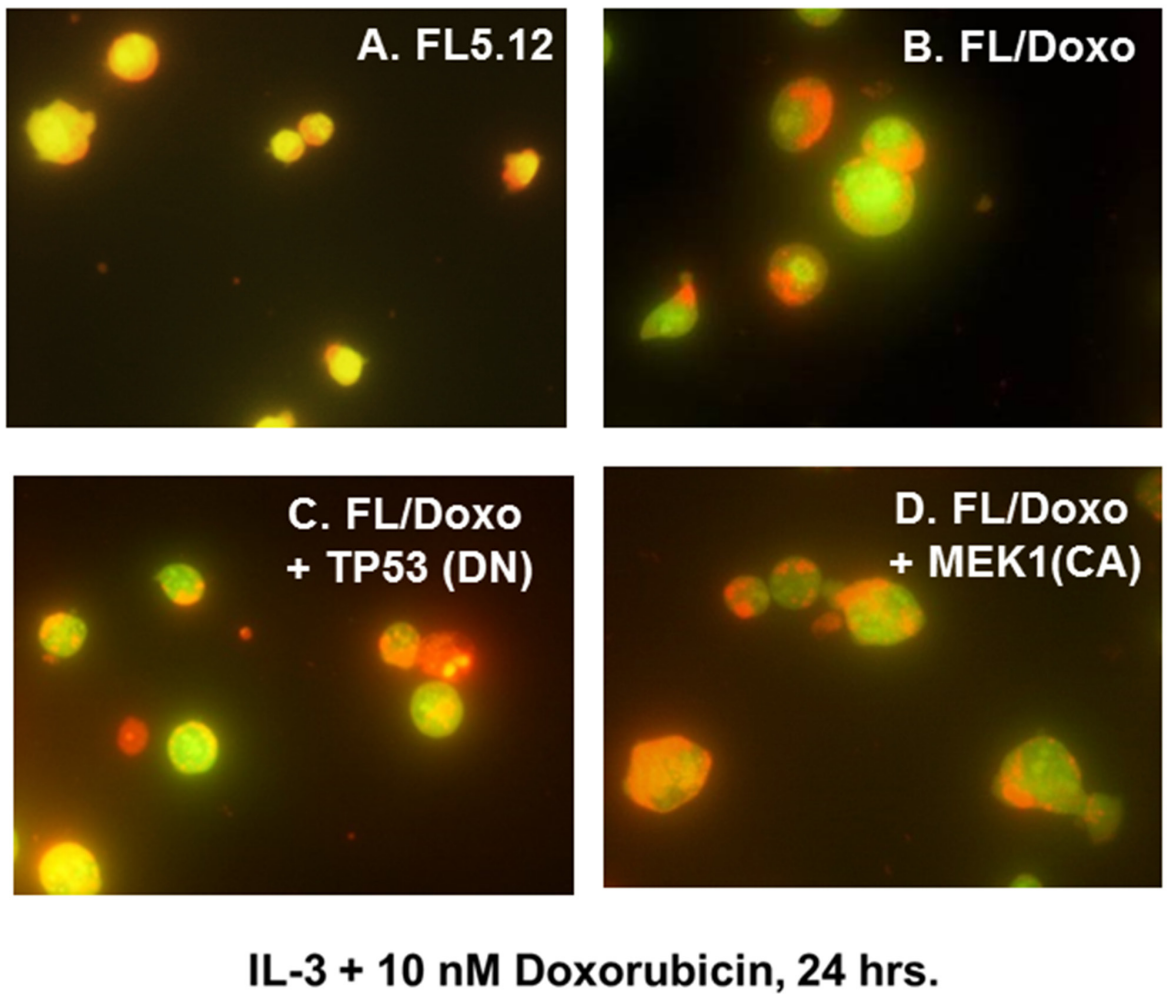
Morphology of FL5.12, FL/Doxo, FL/Doxo + TP53 (DN) and FL/Doxo + MEK1 (CA) cells after staining with acridine orange and ethidium bromide The morphologies of FL5.12 **(Panel A)**, FL/Doxo **(Panel B)**, FL/Doxo + TP53 (DN) **(Panel C)** and FL/Doxo + MEK1 (CA) **(Panel D)** were examined after staining the cells with acridine orange and ethidium bromide. All cells were cultured in the presence of IL-3 + 10 nM doxorubicin before staining and then microscopic examination. A 60X magnification is presented.

**Figure 4:**
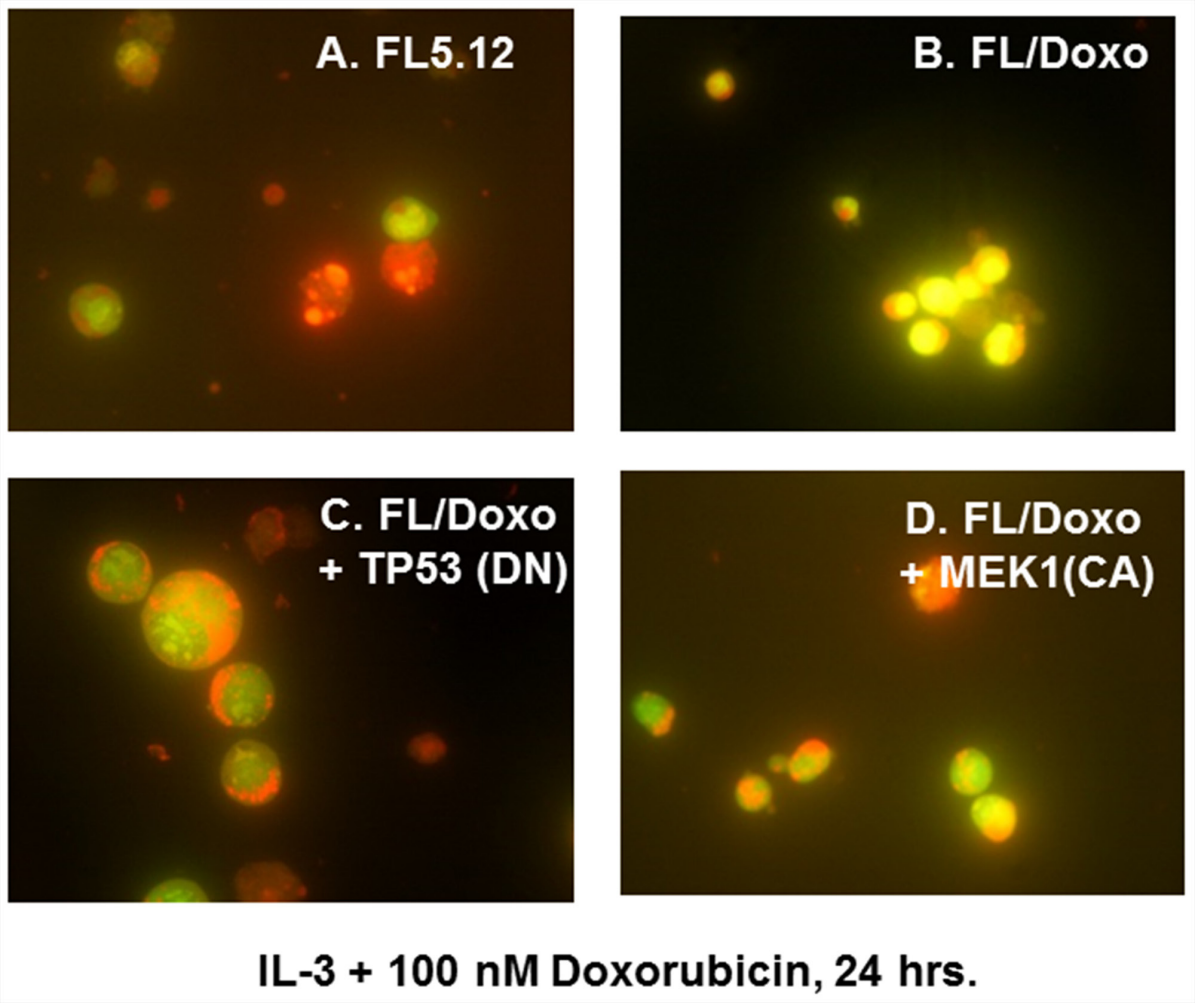
Morphology of FL5.12, FL/Doxo, FL/Doxo + TP53 (DN) and FL/Doxo + MEK1 (CA) cells after staining with acridine orange and ethidium bromide The morphologies of FL5.12 **(Panel A)**, FL/Doxo **(Panel B)**, FL/Doxo + TP53 (DN) **(Panel C)** and FL/Doxo + MEK1 (CA) **(Panel D)** were examined after staining the cells with acridine orange and ethidium bromide. All cells were cultured in the presence of IL-3 + 100 nM doxorubicin before staining and then microscopic examination. A 60X magnification is presented.

Doxorubicin-sensitive FL5.12 and doxorubicin-resistant FL/Doxo, FL/Doxo + TP53 (DN) and FL/Doxo + MEK1 (CA) cells were cultured with IL-3 + 10 nM doxorubicin (Figure 3) or IL-3 + 100 nM doxorubicin (Figure 4) for 24 hrs. Then the cells were stained with acridine orange and ethidium bromide and subsequently photographed. Upon culture with IL-3 + 10 nM doxorubicin, the drug-resistant cell cultures frequently contained larger cells (Figure 3, Panels B, C and D) in comparison to the FL5.12 cells (Figure 3, Panel A).

Upon culture with IL-3 + 100 nM doxorubicin, the drug-sensitive FL5.12 cell cultures frequently contained a large number bright red apoptotic cells (Figure 4, Panel A) in comparison to the FL/Doxo cells (Figure 4, Panel B). This concentration of doxorubicin is above the IC_50_ for FL5.12 cells.

### Drug-resistant FL/Doxo cells display altered drug efflux activity

The abilities of the FL5.12 and FL/Doxo cells to efflux the dye rhodamine 123 (Table 1) were determined by drug efflux assays in the presence and absence of the MDR1 (Pgp, ABCB1) pump inhibitor verapamil or the MRP1 inhibitor MK571. Verapamil is a substrate of ABCB1 (MDR1) [83]. A FACS assay was used to quantitate drug efflux [83].

**Table 1:** Abilities of FL5.12 and FL/Doxo cells to efflux rhodamine 123^1^

Cells	Treatment	T 0hr Point	T 2hr Point	Ratio	Result
FL5.12	-	4.9	3.7		Background
FL5.12	Verapamil	6.8	6		Background
FL5.12	Rhodamine 123	9326.4	4981.1	0.53	Fluorescence decreased with time.
FL5.12	Rhodamine 123 +Verapamil	2042.4	3330.8	1.63	Verapamil inhibiting drug efflux.
FL5.12	MK571	29	21.3		Background
FL5.12	Rhodamine 123 + MK571	6861.2	2740.5	0.4	Verapamil inhibiting drug efflux.
FL/Doxo	-	25.9	23.7		Background
FL/Doxo	Verapamil	24.4	24.7		Background
FL/Doxo	Rhodamine 123	6177.7	3787.5	0.61	Drug fluorescence decreased with time.
FL/Doxo	Rhodamine + Verapamil	2657.7	2590.1	0.97	Fluorescence decreased compared to rhodamine 123-alone treatment. Verapamil blocked further decrease in fluorescence. MDR1 inhibited.
FL/Doxo	MK571	43.3	34.8		Background
FL/Doxo	Rhodamine 123 + MK571	4029.1	598.8	0.14	Fluorescence decreased compared to rhodamine 123- alone treatment. MK571 inhibited drug efflux, MDR1 not inhibited. MDR1 able to pump rhodamine-123 out. 6.7X less fluorescence.

^1^Drug efflux determine by FACS analysis as described by [83].

The background flouresence in FL/Doxo cells (25.9) was higher than that detected in FL5.12 cells (4.9). When the FL/Doxo cells were resuspended in medium containing verapamil, a value of 24.4 was observed while when FL5.12 cells were set up under similar conditions, a value of 6.8 was observed. Higher background levels were observed when the cells were incubated with the MRP1 inhibitor, MK571 as a value of 43.3 was observed with the FL/Doxo cells and 29 was observed with the FL5.12 cells. These values decreased to 34.8 and 21.3 in the FL/Doxo and FL5.12 cells respectively at the T2 hr. time points.

Rhodamine 123 is effluxed by MDR1 [84]. Rhodamine 123 is effluxed to a lesser extent by MRP1 [85]. When FL5.12 cells were cultured in the presence of rhodamine, fluorescence values of 9,326.4 were observed at T0 hr. and 4,981 at the T2 hr. time point (Table 1). A ratio of 0.53. When FL5.12 cells were incubated with rhodamine 123 and verapamil, fluorescence values of 2,042.4 and 3,330.8 were observed (a ratio of 1.63) indicating that verapamil prevented dye efflux at T2 hr. in rhodamine 123-treated FL5.12 cells.

When FL5.12 cells were incubated with MK571 and rhodamine 123 values of 6861.2 were observed at T0 hr. while a value of 2740.5 was observed at the T2 hr. incubation point (a ratio of 0.4). Thus, suppression of MRP1 with MK571 did not prevent total drug efflux.

When FL/Doxo cells were incubated with rhodamine 123, fluorescent values of 6,177.7 and 3,787.5 were observed at the T0 hr. and T2 hr. time points respectively, a ratio of 0.61. When FL/Doxo cells were incubated with rhodamine 123 and verapamil, fluorescent values of 2,657.7 and 2,590.1 (a ratio of 0.97) at the T0 hr. and T2 hr. time points, indicating that verapamil inhibited rhodamine 123 efflux at the T2 hr. time point. More striking results were observed when FL/Doxo cells were incubated with rhodamine 123 and MK571 as fluorescence values of 4,029.1 were observed at the T0 hr. time point and 598.8 were seen at the T2 hr. time point, a ratio of 0.15. Thus, FL/Doxo cells, which express higher levels of MDR1 mRNA transcripts than FL5.12 cells (see Table 2) could efflux more rhodamine 123 than FL5.12 cells (approximately 2.9-fold) upon treatment with the MRP1 inhibitor MK571.

**Table 2:** Levels of MDR1 and MRP1 mRNA transcripts detected after qRT-PCR^1^

Cells Cultured with IL-3					
Cells	MDR1 Transcripts	Fold compared to FL5.12	Cells	MRP1 Transcripts	Fold compared to FL5.12
FL5.12	1.91±0.48	1	FL5.12	211±37.74	1
FL/Doxo	7.17±0.89	3.8X↑	FL/Doxo	249.24±33.57	1.2X↑
FL/Doxo+TP53 (DN)	19.45±2.32	10.2X↑	FL/Doxo+TP53 (DN)	107.15±10.86	2X↓
FL/Doxo+MEK1 (CA)	36.73±2.46	19.2X↑	FL/Doxo+MEK1 (CA)	117.57±7.15	1.8X↓
**Cells Cultured with IL-3 + Doxorubicin**					
**Cells**	**MDR1 Transcripts**	**Fold compared to FL5.12**	**Cells**	**MRP1 Transcripts**	**Fold compared to FL5.12**
FL5.12	0.5±0.42	1	FL5.12	142.75±19.64	1
FL/Doxo	4.28±0.36	8.6X↑	FL/Doxo	181.42±18.26	1.2X↑
FL/Doxo+TP53 (DN)	13.38±1.21	26.7X↑	FL/Doxo+TP53 (DN)	107.15±16.96	1.3X↓
FL/Doxo+MEK1 (CA)	49.84±5.42	99.7X↑	FL/Doxo+MEK1 (CA)	174.4±5.56	1.2X↑

^1^Described in [80].

### Fold changes in MDR1 expression in doxorubicin-resistant FL/Doxo cells when normalized to doxorubicin-sensitive FL5.12 cells

The mRNA expression of MDR1 and MRP1 molecules was examined previously in FL5.12, FL/Doxo, FL/Doxo+TP53(DN) and FL/Doxo+MEK1(CA) cells by q-RT-PCR [80]. The fold differences in the doxorubicin-resistant FL/Doxo in comparison to the doxorubicin-sensitive FL5.12 cells are summarized in Table 2.

The fold increase in MDR1 expression in the cells cultured presence of IL-3 ranged from 3.8-fold in FL/Doxo to 19.2-fold in FL/Doxo+MEK1(CA) cells. In contrast, the levels of MRP1 mRNA transcripts were similar in FL5.12 and FL/Doxo, when the cells were cultured in the presence of IL-3. In contrast, lower levels (2- and 1.8-fold respectively) of MRP1 mRNA transcripts were detected in FL/Doxo+TP53(DN) and FL/Doxo+MEK1(CA) cells respectively than in FL5.12 and FL/Doxo cells.

When the cells were cultured in the presence of IL-3 and 25 nM doxorubicin, the fold increase in MDR1 transcripts in the doxorubicin-resistant cells in comparison with doxorubicin-sensitive FL5.12 cells ranged from between 8.6-fold in FL/Doxo cells to 99.7-fold in FL/Doxo+MEK1(CA) cells. There were large increases in fold increase in MDR1 transcripts in FL/Doxo + MEK1(CA) cells cultured in the presence of doxorubicin. This stems from a 3.8-fold decrease in the levels of MDR1 mRNA transcripts detected in FL5.12 cells cultured in IL-3 and doxorubicin.

Upon culture of the cells in medium containing IL-3 and doxorubicin, levels of MRP1 transcripts were similar in all four cell lines and ranged between 1.2-fold increase in FL/Doxo and FL/Doxo+MEK1(CA) cells and a 1.3-fold decrease in FL/Doxo+TP53(DN) cells in comparison to FL5.12 cells.

### Effects of signal transduction inhibitors on sensitivity to doxorubicin in FL5.12 and drug resistant FL/Doxo derivative cells

**Figure 5:**
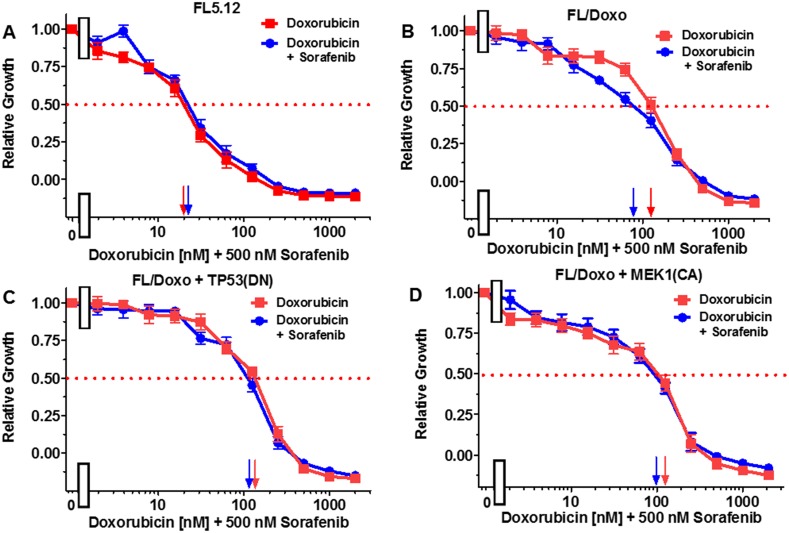
Effects of combination of the multi-kinase inhibitor sorafenib on the doxorubicin IC50 in FL5.12 and FL/Doxo derivative cells The effects of 500 nM sorafenib (solid blue circles) or no sorafenib (solid red squares) on the doxorubicin-sensitivity of: **(Panel A)** FL5.12 cells, **(Panel B)** FL/Doxo cells, **(Panel C)** FL/Doxo + TP53 (DN) cells or **(Panel D)** FL/Doxo + MEK1 (CA) cells were determined by titrating all the cells on the same day. Arrows pointing to the X-axis indicate where the IC_50_ can be estimated. Statistical analysis (unpaired *t* test results) indicated that the two-tailed *P* values for FL/Doxo treated with doxorubicin or doxorubicin and sorafenib in (Panel B) is 0.0046 which is considered to be very statistically significant. These experiments were performed three times with similar results.

**Figure 6:**
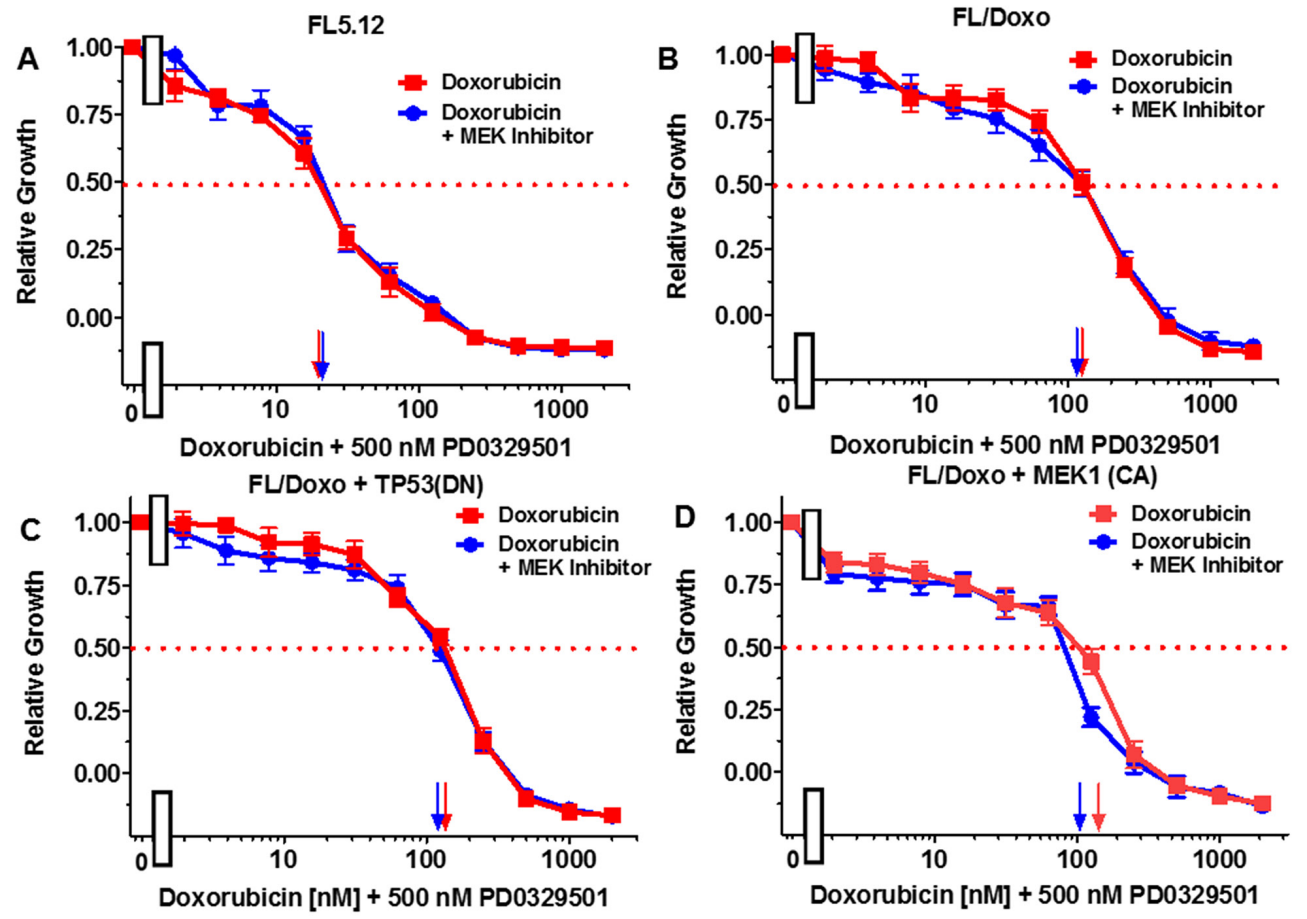
Effects of combination of the MEK1 inhibitor PD0325901 on the doxorubicin IC50 in FL5.12 and FL/Doxo derivative cells The effects of 500 nM PD0325901 (solid blue circles) or no PD0325901 (solid red squares) on the doxorubicin-sensitivity of: **(Panel A)** FL5.12 cells, **(Panel B)** FL/Doxo cells, **(Panel C)** FL/Doxo + TP53 (DN) cells or **(Panel D)** FL/Doxo + MEK1 (CA) cells were determined by titrating all the cells on the same day. Arrows pointing to the X-axis indicate where the IC_50_ can be estimated. Statistical analysis (unpaired *t* test results) indicated that the two-tailed *P* values for FL/Doxo + MEK1 (CA) treated with doxorubicin or doxorubicin and the MEK inhibitor in (Panel D) is 0.0157 and is considered to be statistically significant. These experiments were performed three times with similar results.

**Figure 7:**
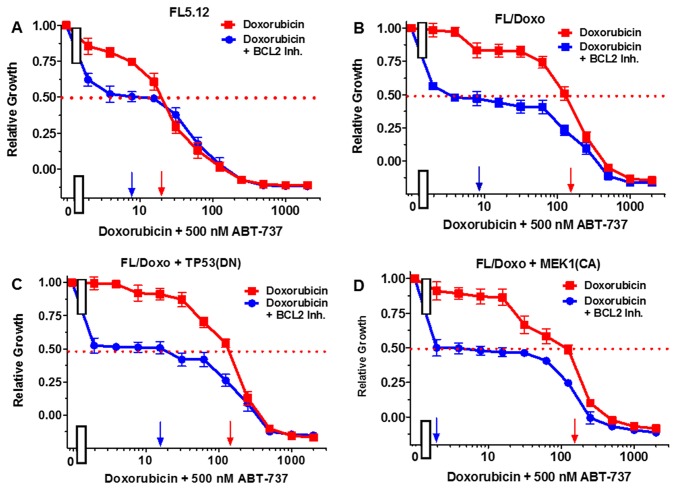
Effects of combination of the BCL2 inhibitor ABT-737 on the doxorubicin IC50 in FL5.12 and FL/Doxo derivative cells The effects of 500 nM ABT-737 (solid blue circles) or no ABT-737 (solid red squares) on the doxorubicin-sensitivity of: **(Panel A)** FL5.12 cells, **(Panel B)** FL/Doxo cells, **(Panel C)** FL/Doxo + TP53 (DN) cells or **(Panel D)** FL/Doxo + MEK1 (CA) cells were determined. Arrows pointing to the X-axis indicate where the IC_50_ can be estimated. Statistical analysis (unpaired *t* test results) indicated that the two-tailed *P* values for the various cells treated with doxorubicin or doxorubicin and the BCL2 inhibitor in: (Panels A-D) are less than 0.0001 which are considered to be extremely statistical significant. These experiments were performed three times with similar results.

The effects of titrating doxorubicin in the presence of the multi-kinase inhibitor sorafenib, which can inhibit RAF and other kinases, the MEK1 inhibitor (PD0325901), and the BCL2 inhibitor ABT737 were examined in Figures 5-7. In all of these graphs, the effects of suboptimal doses of the inhibitors and doxorubicin titrations were determined the same day. They are graphed in this manuscript to illustrate the differences between doxorubicin in the absence of inhibitor and doxorubicin in the presence of the inhibitor in the individual cell lines. In a previous manuscript, the effects of some of these and other inhibitors on the four different FL5.12-derived cell lines were plotted in the same graphs together [80].

FL5.12 cells were cultured in the presence of doxorubicin in the absence and presence of 500 nM of the multi-kinase inhibitor sorafenib (Figure 5, Panel A). An IC_50_ of approximately 20 nM was observed under both conditions. When FL/Doxo cells were cultured in the absence of doxorubicin, an IC_50_ of 120 nM was observed. Upon culture of FL/Doxo cells in the presence of doxorubicin and 500 nM sorafenib, the IC_50_ for doxorubicin was approximately 80 nM (Panel B).

When FL/Doxo + TP53(DN) cells were cultured in the absence of doxorubicin, an IC_50_ of 120 nM was observed. Upon culture of FL/Doxo +TP53(DN) in the presence of doxorubicin and 500 nM sorafenib, the IC_50_ for doxorubicin remained the essentially the same (Panel C).

When FL/Doxo + MEK1(CA) cells were cultured in the absence of doxorubicin, an IC_50_ of 120 nM was observed. Upon culture of FL/Doxo +TP53(DN) in the presence of doxorubicin and 500 nM sorafenib, the IC_50_ for doxorubicin remained the essentially the same (Panel D).

The effects of the MEK1 inhibitor PD0329501 on the doxorubicin IC_50_s were also examined. When FL5.12 cells were cultured in the presence of 500 nM PD0329501 MEK1 inhibitor, the IC_50_ did not change from when they were cultured in the absence of the MEK1 inhibitor (Figure 6, Panel A). Likewise, when FL/Doxo and FL/Doxo + TP53(DN) cells were cultured in the presence and absence of 500 nM PD0325901 MEK inhibitor, the doxorubicin IC_50_s did not change (Figure 6, Panels B and C). In contrast, when FL/Doxo + MEK1(CA) cells were cultured in the presence of 500 nM PD0329501, the doxorubicin IC_50_ was approximately 100 nM while when the cells were cultured in the absence of the MEK1 inhibitor, the doxorubicin IC_50_ was approximately 150 nM (Figure 6, Panel D). Thus, the MEK1 inhibitor treatment reduced the IC_50_ for doxorubicin approximately 1.5-fold in the FL/Doxo + MEK1(CA) cells.

The effects of the BCL2 inhibitor ABT737 on the doxorubicin IC_50_s were also examined (Figure 7). When FL5.12 cells were cultured in the presence of 500 nM ABT 737 inhibitor, the level of growth decreased and the IC_50_ for doxorubicin declined from 20 nM to 8 nM, a 2.5-fold decrease (Figure 7, Panel A). Likewise, when FL/Doxo, FL/Doxo + TP53(DN) and FL/Doxo + MEK1(CA) cells were cultured in the presence and absence of 500 nM ABT-737 (Figure 7, Panel B, C, and D), the level of growth decreased substantially and the doxorubicin IC_50_s decreased from 180 nM to 8 nM (22.5-fold) in FL/Doxo cells, from 130 nM to 18 nM (7.2-fold) in FL/Doxo + TP53(DN) cells and from 160 to 2 nM (80-fold) in FL/Doxo + MEK1(CA) cells. Thus the BCL2 inhibitor decreased the amount of doxorubicin required to achieve the IC_50_.

### Effects of combination of signal transduction and BCL2 inhibitors on FL5.12 and FL/Doxo derivative cells

**Figure 8:**
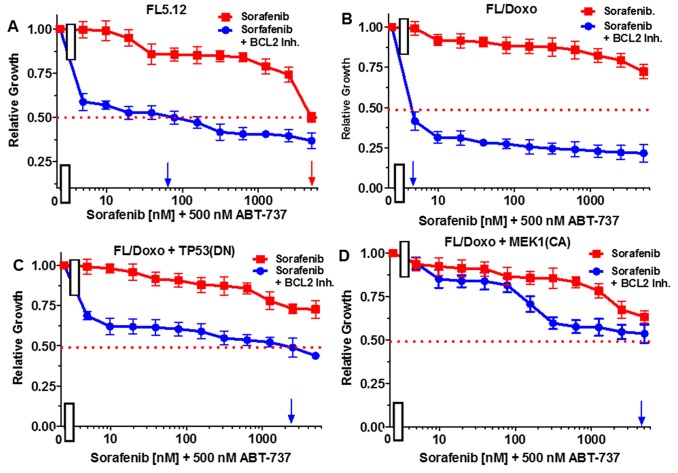
Effects of combination of the BCL2 inhibitor ABT-737 on sorafenib IC50 in FL5.12 and FL/Doxo derivative cells The effects of 500 nM ABT-737 (solid blue circles) or no ABT-737 (solid red squares) on the sorafenib inhibitor-sensitivity of: **(Panel A)** FL5.12 cells, **(Panel B)** FL/Doxo cells, **(Panel C)** FL/Doxo + TP53 (DN) cells or **(Panel D)** FL/Doxo + MEK1 (CA) cells were determined by titrating all the cells on the same day. Arrows pointing to the X-axis indicate where the IC_50_ can be estimated. Statistical analysis (unpaired *t* test results) indicated that the two-tailed *P* values for the various cells treated with doxorubicin or doxorubicin and the BCL2 inhibitor in: (Panel A) is less than 0.0001 which is considered to be extremely statistically significant. These experiments were performed three times with similar results.

The effects of combining sorafenib with the BCL2 inhibitor ABT737 were examined in Figure 8. When FL5.12 cells were cultured in the presence of the multi-kinase sorafenib inhibitor, an IC_50_ of 5,000 nM was observed (Figure 8, Panel A). In contrast, when they were incubated with sorafenib in the presence of 500 nM ABT-737 inhibitor, the level of growth decreased and an IC_50_ of approximately 65 nM (77-fold decrease) was observed.

When FL/Doxo cells were cultured in the presence of sorafenib, an IC_50_ was not obtained even with concentrations up to 5,000 nM (Figure 8, Panel B). In contrast, the level of growth decreased when FL/Doxo cells were cultured with sorafenib in the presence of 500 nM ABT-737 inhibitor and an IC_50_ of approximately 3 nM (approximately a 1,666-fold decrease) with respect to the sorafenib inhibitor was observed.

When FL/Doxo + TP53 (DN) cells were cultured in the presence of sorafenib, the IC_50_ was not obtained even with doses of 5,000 nM doxorubicin (Figure 8, Panel C). In contrast, the level of growth decreased when FL/Doxo + TP53(DN) cells were cultured with sorafenib in the presence of the BCL2 inhibitor and an IC_50_ of approximately 2,000 nM sorafenib was observed. When FL/Doxo + MEK1(CA) cells were cultured in the presence of sorafenib, an IC_50_ was not observed even with concentrations of 5,000 nM sorafenib (Figure 8, Panel D). Addition of the BCL2 inhibitor decreased growth and an IC_50_ of approximately 4,000 nM sorafenib was observed. In the FL/Doxo + MEK1(CA) cells, the decrease was not observed until approximately 100 nM sorafenib, demonstrating that the FL/Doxo + MEK1(CA) cells were more resistant to the BCL2 inhibitor than the other cells.

**Figure 9:**
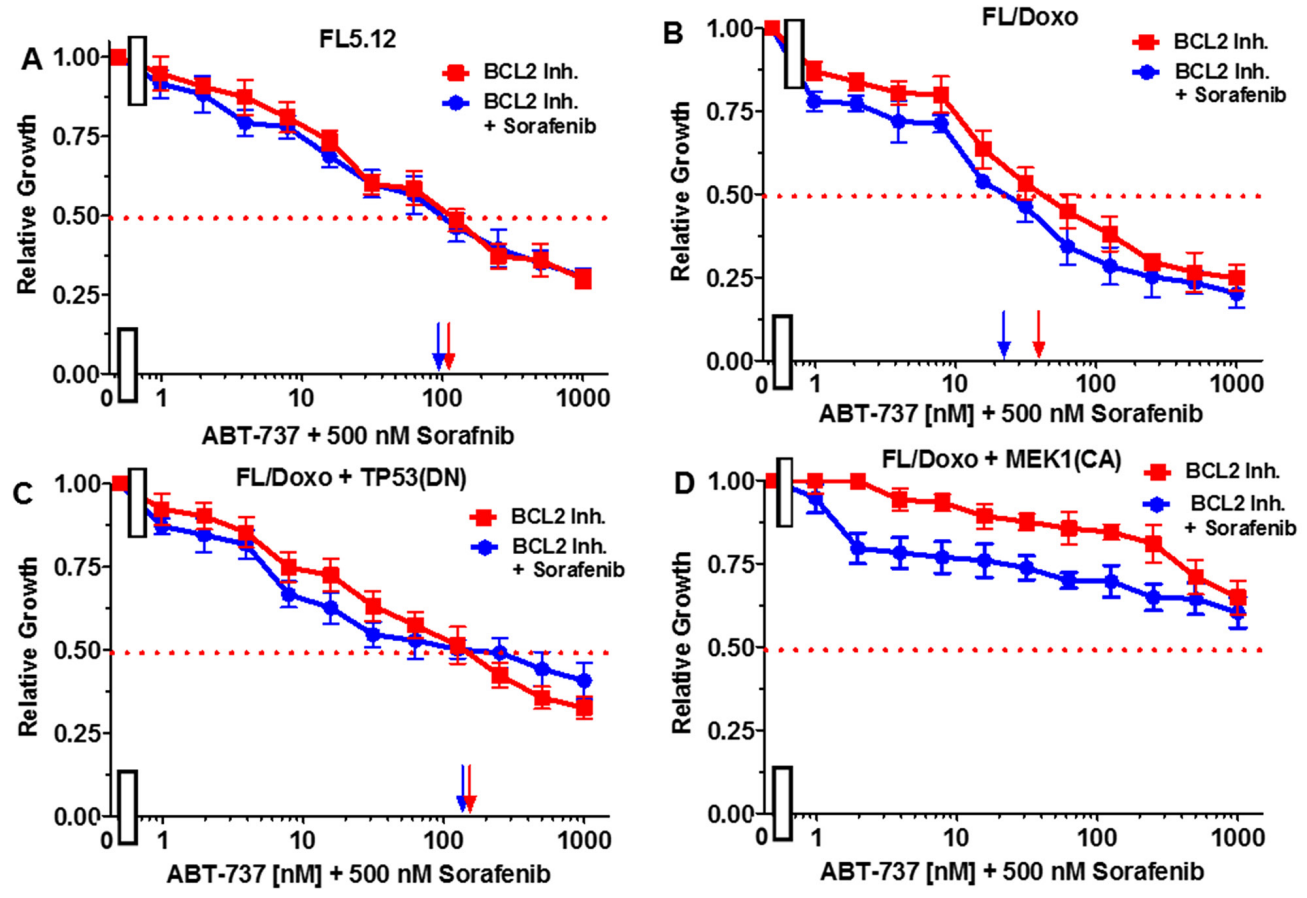
Effects of combination of the multi-kinase inhibitor sorafenib on the BCL2 inhibitor ABT-737 IC50 in FL5.12 and FL/Doxo derivative cells The effects of 500 nM sorafenib (solid blue circles) or no sorafenib (solid red squares) on the BCL2 inhibitor-sensitivity of: **(Panel A)** FL5.12 cells, **(Panel B)** FL/Doxo cells, **(Panel C)** FL/Doxo + TP53 (DN) cells or **(Panel D)** FL/Doxo + MEK1 (CA) cells were determined by titrating all the cells on the same day. Arrows pointing to the X-axis indicate where the IC_50_ can be estimated. Statistical analysis (unpaired *t* test results) indicated that the two-tailed *P* values for the various cells treated with BCL2 inhibitor or BCL2 and sorafenib inhibitors in: (Panel B) is 0.0006 which is considered to be extremely statistically significant, (Panel C) is 0.0290 which is considered to be significant. These experiments were performed three times with similar results.

Essentially the reciprocal experiments were performed. Namely, the effects of titrating cells with the BCL2 inhibitor in the presence and absence of 500 nM sorafenib (Figure 9). Addition of 500 nM sorafenib did not increase the effect of the BCL2 inhibitor on FL5.12 cells (Figure 9, Panel A). When FL/Doxo and FL/Doxo + TP53(DN) cells were treated with 500 nM sorafenib and titrated with the BCL2 inhibitor, the IC_50_s decreased from 40 to 10 nM (4-fold) in FL/Doxo (Panel B) and from 170 to 130 nM (1.3-fold) in FL/Doxo + TP53(DN) cells respectively (Panels B and C). Treatment of the FL/Doxo + MEK1(CA) cells with up to 5,000 nM BCL2 inhibitor did not reach an IC_50_. Combined treatment with different doses of the BCL2 inhibitor and 500 nM sorafenib reduced cell growth but did not reduce the IC_50_ for the BCL2 inhibitor. The experiments mirror the results presented in Figure 8 and document that the FL/Doxo + MEK1(CA) cells were more resistant to the BCL2 inhibitor than the other FL5.12 and FL/Doxo-derived cells.

**Figure 10:**
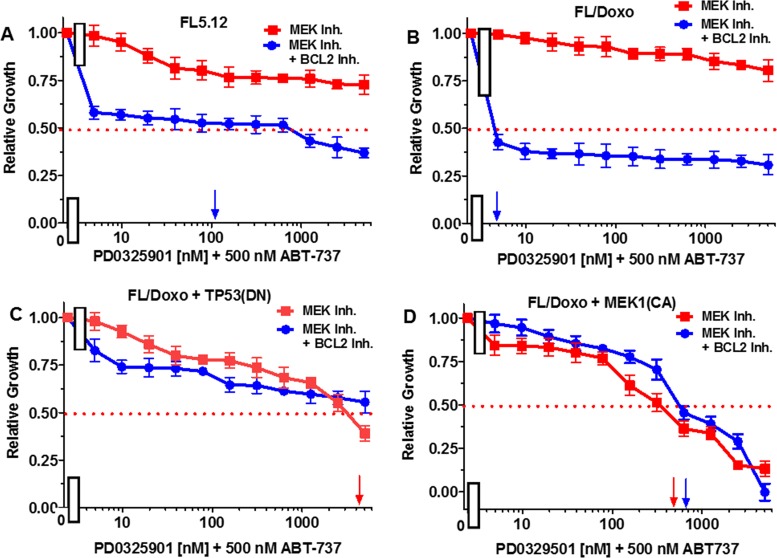
Effects of combination of the BCL2 inhibitor ABT-737 on the MEK inhibitor PD0325901 IC50 in FL5.12 and FL/Doxo derivative cells The effects of 500 nM ABT-737 (solid blue circles) or no ABT-737 (solid red squares) on the MEK inhibitor-sensitivity of: **(Panel A)** FL5.12 cells, **(Panel B)** FL/Doxo cells, **(Panel C)** FL/Doxo + TP53 (DN) cells or **(Panel D)** FL/Doxo + MEK1 (CA) cells were determined by titrating all the cells on the same day in the absence and presence of the BCL2 inhibitor. Arrows pointing to the X-axis indicate where the IC_50_ can be estimated. These experiments were performed three times with similar results.

When FL5.12 cells were cultured in the presence of the PD0329501 MEK1 inhibitor, an IC_50_ was not obtained even with concentrations up to 5,000 nM (Figure 10, Panel A). In contrast, the level of growth decreased when they were cultured with the MEK1 inhibitor in the presence of 500 nM ABT-737 inhibitor and an IC_50_ of approximately 100 nM with respect to the MEK1 inhibitor was observed.

When FL/Doxo cells were cultured in the presence of the PD0329501 MEK1 inhibitor, an IC_50_ was not obtained even with concentrations up to 5,000 nM (Figure 10, Panel B). In contrast, the level of growth decreased when FL/Doxo cells were cultured with the MEK1 inhibitor in the presence of 500 nM ABT-737 inhibitor and an IC_50_ of approximately 3 nM with respect to the MEK1 inhibitor was observed.

When FL/Doxo + TP53 (DN) cells were cultured in the presence of the PD0329501 MEK1 inhibitor, an IC_50_ of approximately 3,000 nM was observed (Figure 10, Panel C). The level of growth decreased slightly when FL/Doxo + TP53(DN) cells were cultured with the MEK1 inhibitor in the presence of 500 nM ABT-737 inhibitor, but the IC_50_ for the MEK1 inhibitor was not obtained even with doses of 5,000 nM PD0329501.

When FL/Doxo + MEK1(CA) were cultured in the presence of the PD0329501, an IC_50_ of approximately 400 nM MEK1 inhibitor was observed (Figure 10, Panel D). In contrast, the addition of 500 nM BCL2 inhibitor did not decrease the concentration of the MEK1 inhibitor required to reach the IC_50_, if anything, it antagonized slightly with the MEK1 inhibitor in FL/Doxo + MEK1(CA) cells.

**Figure 11:**
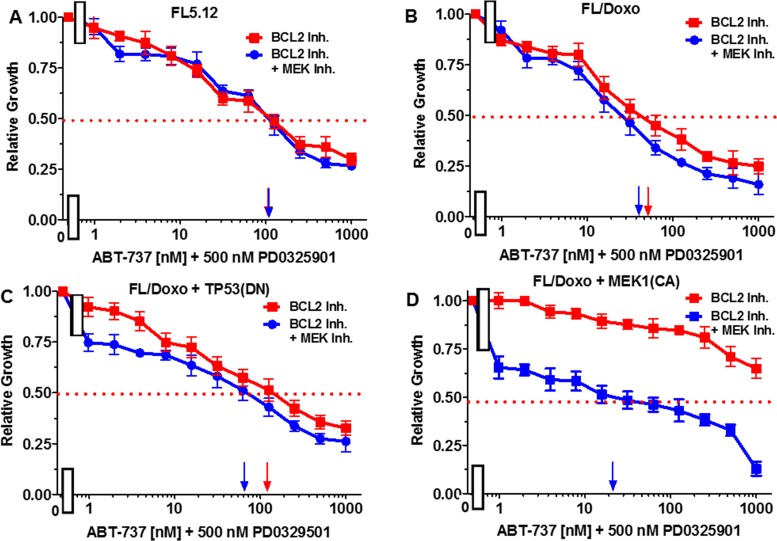
Effects of combination of the MEK inhibitor PD0325901 on the BCL2 inhibitor ABT-737 IC50 in FL5.12 and FL/Doxo derivative cells The effects of 500 nM PD0325901 (solid blue circles) or no PD0325901 (solid red squares) on the BCL2 inhibitor-sensitivity of: **(Panel A)** FL5.12 cells, **(Panel B)** FL/Doxo cells, **(Panel C)** FL/Doxo + TP53 (DN) cells or **(Panel D)** FL/Doxo + MEK1 (CA) cells were determined by titrating all of the cells on the same day in the absence and presence of the MEK inhibitor. Arrows pointing to the X-axis indicate where the IC_50_ can be estimated. Statistical analysis (unpaired *t* test results) indicated that the two-tailed *P* values for the various cells treated with BCL2 or BCL2 and MEK1 inhibitor in: (Panel B) is 0.0073 which is considered to be very statistically significant, (Panel C) is less than 0.0011 which is considered to be very statistically significant. These experiments were performed three times with similar results.

Essentially the reciprocal experiments were performed. Namely, the effects of titrating cells with the BCL2 inhibitor in the presence and absence of the 500 nM MEK1 inhibitor were determined (Figure 11). Addition of 500 nM MEK inhibitor did not reduce the effect of the BCL2 inhibitor on FL5.12 cells (Figure 11, Panel A). When FL/Doxo and FL/Doxo + TP53(DN) cells were treated with 500 nM MEK inhibitor and titrated with the BCL2 inhibitor, the IC_50_s decreased from 55 to 45 nM (1.2-fold) in FL/Doxo (Panel B) and from 120 to 70 nM (1.7-fold) in FL/Doxo + TP53(DN) cells respectively (Panels B and C). In contrast, a more significant result was observed in FL/Doxo + MEK1(CA), the IC_50_ for the BCL2 inhibitor dropped from greater than 5,000 nM to approximately 20 nM. The experiments mirror the results presented in the previous figure and document that the FL/Doxo + MEK1(CA) cells are sensitive to the MEK1 inhibitor while resistant to the BCL2 inhibitor. In contrast the FL/Doxo, FL/Doxo + TP53(DN) and FL5.12 cells are more sensitive to the effects of the BCL2 inhibitor than the MEK1 inhibitor.

### Drug-resistant FL/Doxo cells displayed increased levels of side-population positive cells

The presence of side-population positive cells in the FL5.12 and different FL/Doxo cells was examined. Side-population positive cells is often associated with drug-resistance and CSCs [86]. MDR1 expression has been shown to be important in glioma CSCs [87]. Verapamil has been shown to inhibit tumor progression by targeting the side population of certain types of cancer cells [88].

**Figure 12:**
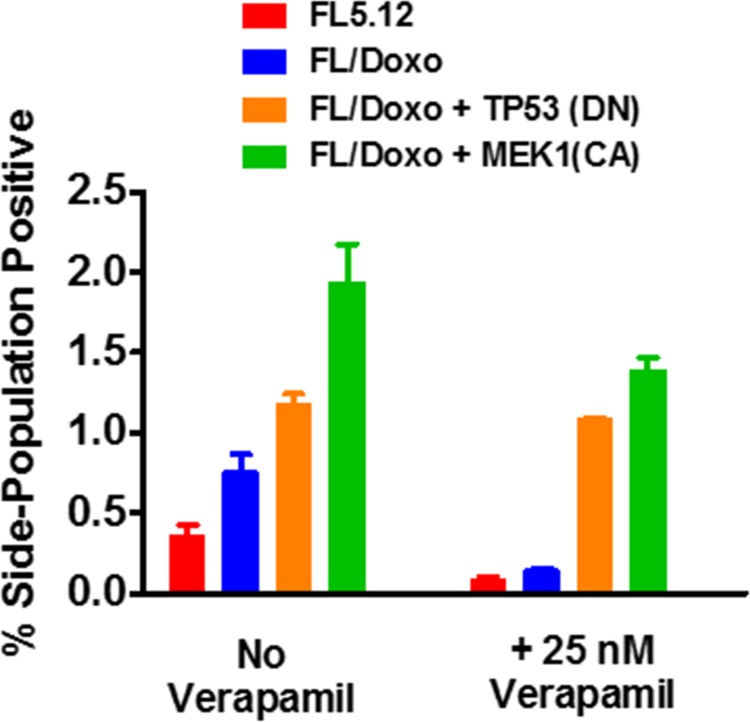
Presence of side-positive cells in FL5.12, FL/Doxo, FL/Doxo + TP53 (DN) and FL/Doxo + MEK1 (CA) cells The presence of side-positive cells was determined by FACS analysis. In some cases, the cells were cultured with 25 nM verapamil. Statistical analysis (unpaired *t* test results) indicated that the two-tailed *P* value for the FL5.12 (no verapamil) and FL/Doxo (no verapamil) is 0.0195 which is considered to be statistically significant. Statistical analysis (unpaired *t* test results) indicated that the two-tailed *P* value for the FL5.12 (plus verapamil) and FL/Doxo (plus verapamil) is 0.2879 which is not statistically significant. Statistical analysis (unpaired *t* test results) indicated that the two-tailed *P* value for the FL5.12 (no verapamil) and FL/Doxo + TP53(DN) (no verapamil) is 0.0005 which is considered to be extremely statistically significant. Statistical analysis (unpaired *t* test results) indicated that the two-tailed *P* value for the FL5.12 (plus verapamil) and FL/Doxo + TP53 (DN) (plus verapamil) is 0.0001 which is considered extremely statistically significant. Statistical analysis (unpaired *t* test results) indicated that the two-tailed *P* value for the FL5.12 (no verapamil) and FL/Doxo + MEK1(CA) (no verapamil) is 0.0013 which is considered to be very statistically significant. Statistical analysis (unpaired *t* test results) indicated that the two-tailed *P* value for the FL5.12 (plus verapamil) and FL/Doxo + MEK1 (CA) (plus verapamil) is 0.0002 which is considered extremely statistically significant.

Side-population positive cells were detected as we and others have previously described [88–90]. Statistically significant higher levels of side-population positive cells were detected by FACSs analysis in FL/Doxo than in FL5.12 cells (Figure 12). Approximately 1.9-fold more side population positive cells were detected in FL/Doxo cells than in FL5.12 cells. The detection of side population positive cells was prevented with the FL5.12 and FL/Doxo cells were treated with 25 nM verapamil which inhibits MDR1.

The presence of side-population positive cells in the FL/Doxo + TP53 (DN) and FL/Doxo + MEK1 (CA) cells was also examined in the same experiments (Figure 12). Increased levels of side-population positive cells were detected in the FL/Doxo + TP53 (DN) and FL/Doxo + MEK1 (CA) cells than in either FL/Doxo or FL5.12 cells. Approximately 3- and 4.5-fold more side-positive cells were detected in FL/Doxo + TP53 (DN) and FL/Doxo + MEK1 (CA) cells than in FL5.12 cells. In contrast to the results observed with FL/Doxo and FL5.12 cells, the percentage of side-population positive FL/Doxo + TP53 (DN) and FL/Doxo + MEK1 (CA) cells was not as inhibited in FL/Doxo and FL5.12 cells after 25 nM verapamil treatment.

## DISCUSSION

The cytokine IL-3 was protective in FL5.12 cells against the effects of doxorubicin. When FL5.12 cells were cultured in the absence of IL-3, the IC_50_ was approximately 6.7-fold less than when they were grown in the presence of IL-3. FL5.12 cells will undergo apoptosis when they are cultured in the absence of IL-3 [75, 79, 81]. Upon their incubation in the absence of IL-3 in the presence of doxorubicin, their growth is even more inhibited than when they are cultured in the presence of IL-3 and doxorubicin. Doxorubicin-resistant FL/Doxo cells often were larger than doxorubicin-sensitive FL/Doxo cells and acridine orange staining has indicated that they are often multi-nucleate [81].

Doxorubicin induced more apoptosis in the doxorubicin-sensitive cells at lower doxorubicin concentrations than in the doxorubicin-resistant cells [79]. Similar results were observed with daunorubicin and paclitaxel with the FL5.12 and FL/Doxo cells [79]. Previously we demonstrated that there was decreased caspase activation (caspases 3, 8 and 10) in the doxorubicin-resistant FL/Doxo cells than in the doxorubicin-sensitive FL5.12 cells after doxorubicin treatment [79]. In this current manuscript, we have focused on drug efflux, the effects of the MDR1 and the MRP1 inhibitors on drug efflux, drug transporter gene expression, and the effects of signal transduction and BCL2 inhibitors on growth, and the presence of side-population positive cells in drug-sensitive and drug-resistant cells.

Both FL5.12 and FL/Doxo cells could readily efflux rhodamine 123. In both FL5.12 and FL/Doxo cells, the level of fluorescence detected after rhodamine 123 staining decreased after 2 hrs. on ice. The efflux of rhodamine 123 was inhibited in both FL5.12 and FL/Doxo cells after verapamil treatment. In contrast, after treatment with the MK571 MRP1 inhibitor, the efflux of rhodamine 123 was not as inhibited. The FL/Doxo cells appeared to efflux approximately 2.9-fold more rhodamine 123 than the FL5.12 cells when they were treated with the MK571 MRP1 inhibitor.

The levels of mRNAs of MDR1 and MRP1 have been quantitated by qRT-PCR [80]. In this current manuscript we discuss the fold differences in mRNA expression. The cell lines with the highest degree of drug-resistance, FL/Doxo+TP53(DN) and FL/Doxo+MEK1(CA) expressed approximately 10-100-fold more MDR1 mRNA transcripts than in the drug-sensitive FL5.12 cells. FL/Doxo cells expressed approximately 4-fold more MDR1 transcripts than FL5.12 cells. The fold differences in MDR1 expression between the doxorubicin-sensitive FL5.12 cells and doxorubicin-resistant FL/Doxo and derivative cells increased when the cells were cultured in the presence of doxorubicin as the levels of MDR1 mRNAs detected in the FL5.12 cells decreased.

In contrast, 2- to 4-fold less BAX, BIM, BCL2 and BIM mRNAs and between 1.3 to 2-fold less MCL1 mRNA transcripts were detected in the cells with the highest-degree of drug-resistance, FL/Doxo+TP53(DN) and FL/Doxo+MEK1(CA) than in the drug-sensitive FL5.12 cells [80]. The FL/Doxo cells expressed 2-4-fold less BCL2 mRNA transcripts than the FL5.12 cells. The FL/Doxo cells were very sensitive to the BCL2/BCLXL inhibitor ABT-737 [80]. Our results with the expression of the anti-apoptotic BCL2 and BCLXL mRNA expression were interesting as the cells that were more doxorubicin-sensitive expressed more of these transcripts as detected by q-RT-PCR. Thus, in our system, doxorubicin-resistance is associated with lower expression of BCL2 and BCLXL RNA expression. Previously we determined that ectopic expression of BCL2 could enhance the ability of the RAF and MEK1 oncoproteins to abrogate the cytokine-dependence of IL3-dependent cells [91, 92].

Our results indicate the MEK1(CA) and the TP53(DN) genes can have effects on both the expression of MDR1 and the presence of side-population positive cells. These results are important as these characteristics are often linked with CSC. CSCs have been detected in almost every cancer type. Novel methods to target CSCs are under intense investigation. Other studies have shown the side-population positive cells includes CSCs [93]. Chemotherapeutic drug-resistance is often associated with drug transporters, side-population positive and CSCs [86]. Other investigators have documented the sensitivity of other cancer cells to verapamil treatment and their enhanced abilities to form colony *in vitro* and tumors in animals [94]. In the above study, the presence of side-population positive AsPC1 pancreatic cancer cells was not eliminated completely after verapamil treatment. These and our results indicate that there may be other drug transporters not inhibited by verapamil that are present in some of the cells which contribute to their side-population positive cells and drug resistance. Alternatively, the concentration of verapamil used may not have been sufficient to inhibit all the MDR1 molecules expressed in the drug-resistant cells.

An effect of sorafenib on the sensitivity to doxorubicin was observed in FL/Doxo but not in the other FL/Doxo derivative or parental FL5.12 cells. In most experiments, the FL/Doxo are more sensitive to doxorubicin, than either FL/Doxo + TP53(DN) or FL/Doxo + MEK1(CA) cells. In these studies, addition of 500 nM PD0325901 MEK inhibitor only affected the doxorubicin IC_50_ in FL/Doxo + MEK1(CA) cells. In contrast, addition of 500 nM ABT-737 BCL2 inhibitor reduced both the level of cell growth and the doxorubicin IC_50_ in FL5.12, FL/Doxo and FL/Doxo +TP53(DN) and FL/Doxo + MEK1(CA) cells. Thus, the ABT-737 BCL2 inhibitor could reduce the IC_50_ for doxorubicin in all cell lines. In contrast, the FL/Doxo+ MEK1(CA) cells were relatively resistant to the BCL2 inhibitor by itself or in combination with certain signal transduction inhibitors.

The PI3K/PTEN/AKT/mTORC1/GSK-3 pathway regulates the expression of various drug transporter molecules [95–97]. The PI3K/PTEN/AKT/mTORC1/GSK-3 pathway can induce multidrug-resistance in AML cells that were co-cultured with stromal cells [98].

Various signaling pathways have been implicated in drug resistance, both resistance to classical chemotherapeutic drugs, as well as, targeted therapeutics. Often resistance is associated with CSCs and CSCs frequently display altered activity of proteins which transport drugs. Our studies have documented the importance of activated MEK1 in drug-resistance and side-population positive cells in hematopoietic cells.

Parental FL5.12 and FL/Doxo + TP53(DN) cells were also sensitive to the BCL2 inhibitor, but the FL/Doxo + MEK1(CA) cells were relatively resistant to the BCL2 inhibitor ABT-737. It is possible that the activated MEK1 present in the FL/Doxo + MEK1(CA) cells, activated ERK1,2 which phosphorylated a BCL2 family member which prevented the effects of the BCL2 inhibitor. Importantly, the doxorubicin-sensitivity of all the cell lines examined could be reduced upon treatment with the BCL2 inhibitor.

Various BCL2 family members have been observed to be phosphorylated by RAF/MEK/ERK signaling which could have various effects on apoptosis, cellular proliferation, prevention of apoptosis and drug-resistance [99–106]. Interactions between RAF/MEK/ERK and BCL2 family members have been shown to abrogate the cytokine-dependency of certain hematopoietic cells [91, 92]. BCL2 inhibitors increased the sensitivity of the cells to doxorubicin in the current study. Inhibition of RAF/MEK/ERK signaling by sorafenib also had some effects on the doxorubicin-sensitivity of FL/Doxo cells.

Mutation of TP53 may also contribute to drug-resistance and CSCs. We have demonstrated that introduction of the DN-TP53 gene into FL/Doxo cells resulted in more drug- resistance and increased side-population positive cells. Inheritance of DN-TP53 also resulted in decreased anti-apoptotic and pro-apoptotic BCL2 family expression. In contrast, inheritance of DN-TP53 resulted in increased levels of beta2-microglobulin mRNA transcripts [80]. Interestingly, FL/Doxo + TP53 (DN) cells were very sensitive to the BCL-2 inhibitor, ABT-737. These cells were relatively resistant to MEK1 inhibitor PD0329501. Addition of 500 nM MEK inhibitor to cells titrated with the BCL2 inhibitor decreased the IC_50_ required for the BCL2 inhibitor less than 2-fold.

Similar experiments performed with the combination of the MEK1 inhibitor and titration with the BCL2 inhibitor in FL/Doxo + MEK1(CA) cells reduced the IC_50_ for the amount of BCL2 inhibitor approximately 500-fold. While FL/Doxo + TP53(DN) cells expressed high levels of MDR1 as did FL/Doxo + MEK1(CA) cells, but they differed in their sensitivities to BCL2 and MEK1 inhibitors.

In conclusion, our studies document the effects of signaling pathways and TP53 on the drug-resistance of hematopoietic cells. By using a single parental cell line and three drug resistant derivatives of it which either have or lack TP53 (DN) or MEK1(CA), we can identify some of the key components in resistance to doxorubicin a common, clinically used chemotherapeutic drug. Namely increased expression of MDR1 and altered BCL2 family member expression which contribute to altered sensitivity to doxorubicin as well as targeted therapeutics. Our results may have clinical significance as some of the drugs and signal transduction inhibitors and their next generation counter-parts are being used to treat various cancers.

## MATERIALS AND METHODS

### Cells and tissue culture conditions

FL5.12, FL/Doxo, FL/Doxo + TP53 (DN) and FL/Doxo + MEK1 (CA) were derived as described [72, 79–81]. The cells were cultured in RPMI containing antibiotics, l-glutamine 10% fetal bovine serum and 10% WEHI-3B supernatant as a source of murine IL-3 as described [78–81].

### Treatment of cells with signal transduction inhibitors and doxorubicin

FL5.12, FL/Doxo, FL/Doxo + TP53 (DN) and FL/Doxo + MEK1 (CA) were titrated with the different signal transduction inhibitors and doxorubicin as described [79–81]. Statistical analysis was performed using GraphPad Prism. MTT proliferation assays were performed as described [79–81].

### Drug efflux assays

Drug Efflux assays were performed by FACS analysis as described previously [83].

### Side population analysis

Side population analysis was performed as described [88–90]. Briefly, FL5.12, FL/Doxo, FL/Doxo + TP53(DN) and FL/Doxo + MEK1(CA) Cells were resuspended at 1 × 10^6^/mL in RPMI 1640 with 10% FBS and 10% WEHI-3B supernatant in the presence and absence of 25 nM verapamil. Hoechst 33342 dye (Sigma-Aldrich, Saint Louis, Missouri) was added to a final concentration of 5 μg/mL. The cells were incubated at 37°C for 90 minutes. Cells were then centrifuged and the pellet resupended in cold PBS containing 2% FBS. Cells were counterstained with propidum iodide (10 μg/ml). Hoechst dye was excited at 355 nM. The side-population positive analysis was then performed as described [88–90] on a BD FACSVantage. The side-population population, essentially disappeared in FL5.12 and FL/Doxo cells when they were cultured in the presence of verapamil. Experiments were set up in triplicate in 6-well plates.

### Quantitative RT-PCR and normalization to FL5.12 cells

TaqMan Gene Expression assays were described previously [80]. Levels of mRNA transcripts were normalized to the levels of the particular gene transcripts detected in FL5.12 cells which was set at 1.
